# Development of evidence-based practice in occupational health services in Sweden: a 3-year follow-up of attitudes, barriers and facilitators

**DOI:** 10.1007/s00420-017-1200-8

**Published:** 2017-02-15

**Authors:** Elisabeth Björk Brämberg, Teresia Nyman, Lydia Kwak, Akbar Alipour, Gunnar Bergström, Liselotte Schäfer Elinder, Ulric Hermansson, Irene Jensen

**Affiliations:** 1 Unit of Intervention and Implementation Research for Worker Health, Institute of Environmental Medicine, Karolinska Institutet, Nobels väg 13, 171 77 Stockholm, Sweden; 2 Unit of Ergonomics, School of Technology and Health, KTH Royal Institute of Technology, Stockholm, Sweden; 3 Department of Medical Sciences, Occupational and Environmental Medicine, Uppsala University, Uppsala, Sweden; 4 Storvretens Primary Health Clinic, Stockholm, Sweden; 5 Centre for Occupational and Environmental Medicine, Stockholm County Council, Stockholm, Sweden; 6 Department of Public Health Sciences, Karolinska Institutet, Stockholm, Sweden; 7 Department of Clinical Neuroscience, Karolinska Institutet, Stockholm, Sweden

**Keywords:** Evidence-based practice, Quality of occupational health services, Scientific knowledge, Clinical expertise, Questionnaire, Interviews

## Abstract

**Purpose:**

The Swedish government initiated an investigation of how to secure and develop the competence of the occupational health services. The primary aim of the present study was to investigate whether the development of evidence-based practice (EBP) in the Swedish occupational health services in relation to attitudes, knowledge and use improved during the first 3 years of the government’s initiative.

**Methods:**

The study has a mixed methods design combining questionnaires and interviews with data collection at baseline and at 3-year follow-up.

**Results:**

The response rate was 66% at baseline and 63% at follow-up. The results show that practitioners’ knowledge of EBP was moderate at baseline and improved at follow-up (*p* = 0.002; 95% CI 0.01; 0.21). Practitioners experienced lower levels of organizational and managerial support for EBP at follow-up (*p* < 0.001; 95% CI 0.18; 0.38). The results revealed that managers viewed responsibility for implementing EBP as a matter for individual practitioners rather than as an organizational issue.

**Conclusions:**

Occupational health service managers and practitioners are generally positive to EBP. However, the findings emphasize the need to educate managers in how to support EBP at the organizational level by creating an infrastructure for EBP in the OHS.

## Introduction

The concept of evidence-based practice (EBP) (Sackett et al. 1996) was introduced to health care as a means of linking research findings and clinical practice (Andermann et al. 2016; Powell et al. 2015). There is now extensive literature on the implementation of EBP in different health care settings and disciplines (e.g., Jones et al. 2015; Jun et al. 2016; Lau et al. 2016; Verbeek et al. 2002; Zwolsman et al. 2012) as well as using EBP for policies and decision-making in health care (Innvaer et al. 2002; Vogel et al. 2013). However, research is limited with regard to the occupational health services (OHS). Previous research into the implementation of EBP in OHS has focused on clinical guidelines (Hulshof and Hoenen 2007; Lugtenberg et al. 2016) and the use of physical rehabilitation (Beattie et al. 2013). The use of evidence has also been associated with improved clinical decision-making and patient care (Schaafsma et al. 2007). However, the implementation of EBP is a complex process with a diversity of influencing factors, such as the evidence itself, the clinician’s attitude towards the evidence and the implementation strategies used (Zwerver et al. 2013). Barriers to implementation can be knowledge-related, such as lack of awareness or familiarity, lack of skills for formulating literature search questions, lack of effective search strategies and lack of understanding of how to translate findings into clinical practice. Barriers can also be related to attitude, for example lack of agreement or motivation, or to client expectations (Joosen et al. 2015; van Dijk et al. 2010; Verweij et al. 2012). Barriers can also be explained by organizational factors such as insufficient time (van Dijk et al. 2010). Facilitating factors have been described as ranging from the organizational context, such as research councils, libraries, access to occupational health literature databases and legal and administrative regulations, to the clinical (implementation) context, such as education and training and access to online libraries and full-text articles. Management support and economic resources facilitate the use of evidence (Hugenholtz et al. 2009).

Several efforts have been made to improve the scientific basis of the OHS and increase the use of best evidence (cf. Hulshof and Hoenen 2007; Joosen et al. 2015; Kinnunen-Amoroso 2013). The Cochrane work group has conducted a number of systematic reviews, summarizing the body of knowledge-related occupational health and safety (e.g., Klimas et al. 2014; Ruotsalainen et al. 2015). In Sweden, the government decided to initiate an investigation into how to secure and develop the competence of the OHS. This initiative resulted in the funding of two 6-year research programs (2011–2016), which included the funding of the first Swedish professorship in OHS research. The first 3-year period has largely focused on building up a partnership organization engaging researchers, OHS services and labor market parties; initiating a competence center and developing practice guidelines (Hermansson and Occupational Health Guideline Group 2016; Jensen and Occupational Health Guideline Group 2014, 2015; Kwak and Occupational Health Guideline Group 2015).

Although there are similarities between OHS and other health care disciplines, the clinical questions and professional focus of the OHS mainly concern work environment risks and the protection and promotion of workers’ health rather than the treatment of disease. The aim of the OHS is to prevent work-related illness and protect and promote workers’ health and work ability (Heselmans et al. 2010; Kwak et al. 2014). In Sweden, the OHS is not part of the state-funded health care system; it operates on the open market. In line with the Work Environment Act (Swedish Work Environment Authority 2014), an employer must ensure that the OHSs required by the working conditions of that particular workplace are available. The OHS is defined as “an independent expert resource in the field of occupational safety and health. Occupational health services shall in particular work for the prevention and elimination of health risks at workplaces, and shall have the competence to identify and describe associations between the working environment, organization, productivity and health”. According to the Swedish Work Environment Authority, 62% of the Swedish working population has access to an OHS. Approximately 28% of the male workforce and 19% of the female workforce consulted the OHS in 2013 [The Swedish Work Environment Authority (Arbetsmiljöverket) 2014]. The OHS in Sweden employs about 4700 persons; the main professional categories are nurses, physicians, physiotherapists, ergonomists, psychologists, behavioral therapists, environmental engineers and administrative staff.

The use of EBP improves clinical decision-making and patient-related outcomes (Franco and Grandi 2008; Schaafsma et al. 2007). EBP also contributes to the quality of the service and the independence of practitioners in the OHS (Ghafur et al. 2013). However, the use of evidence is known to be facilitated or hindered by organizational and individual factors (Lugtenberg et al. 2016). Hence, understanding the attitudes and skills of OHS practitioners, their use of EBP as well as perceived barriers and facilitators is a critical step in advancing EBP and increasing the uptake of research into OHS.

## Aim

The primary aim of the present study was to investigate whether the development of evidence-based practice in relation to attitudes, knowledge and use in the Swedish occupational health service improved during the first 3 years of the government initiative. Our aim was also to investigate facilitators of and barriers to the implementation of EBP.

## Methods

### Study design

The study has a prospective mixed method design (Creswell 2009), using both questionnaires and interviews. At baseline (2011) and follow-up (2014) data were collected by means of questionnaires answered by OHS practitioners. Qualitative data were collected at follow-up by means of semi-structured interviews with OHS managers.

### Study population—questionnaire

**Fig. 1:**
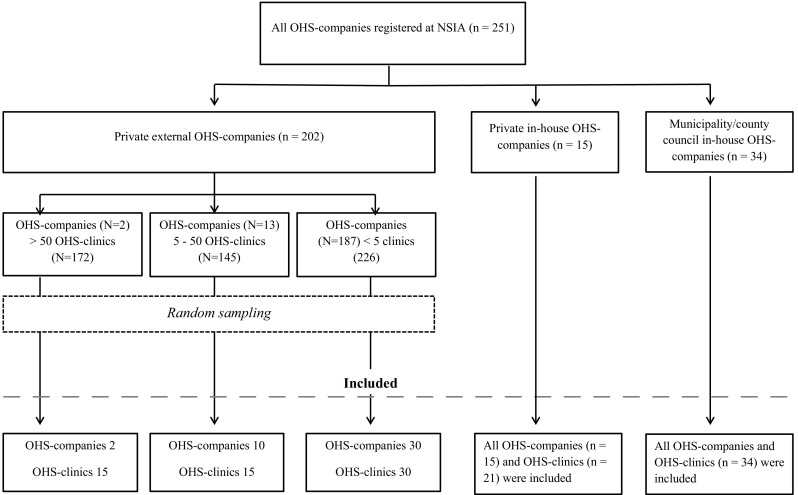
Flow chart sampling procedure

Based on the register of the Swedish association for OHS, the population consisted of 251 OHS companies operating 598 clinics (total employees ≈ 4,700, including administrative personnel) in 2011. A random sample of these 251 OHS companies was selected using a stepwise approach (Fig. 1). In order to have an equal distribution with regard to type of company (in-house, external, private, municipality/county council owned), the OHS were divided into three groups in the first step: (1) private external OHS companies, i.e., privately-owned companies providing services to several customer companies; (2) private in-house OHS companies, i.e., owned by large companies, providing services mainly to their own employees; (3) municipality/county council in-house OHS companies, i.e., owned by municipalities or county councils, providing services to their own employees.

Of the 251 OHS companies, 202 (comprising 543 OHS clinics) were private external and dominated by three very large OHS enterprises running a large number of clinics. Consequently, in the second step, private external OHS companies were divided into three subgroups based on the number of OHS clinics they ran: (1) OHS companies with more than 50 OHS clinics; (2) OHS companies with 5–50 OHS clinics and (3) OHS companies with less than five clinics. The number of companies in the three subgroups was 2, 13 and 187, respectively, with 172, 145 and 226 clinics. Given the varying number of clinics in each subgroup, a random selection from each subgroup was made to obtain 15 clinics each from subgroup 1 and 2, and 30 clinics from subgroup 3. Each OHS clinic was treated as a separate functional unit in the sample. The random sampling was performed without replacement using an SPSS random sample generator. All private in-house and municipality/county council in-house OHS companies were included in the sample.

The selection gave 91 OHS companies covering 115 OHS clinics, all of which were invited to participate. The same sample was contacted 3 years later, in 2014. Ten of the previously included OHS no longer existed as a result of reorganization or merger. The 2014 sample therefore consisted of 71 OHS companies covering 105 OHS clinics.

An electronic link to the web-based questionnaire was sent to all practitioners (*n* = 853) at the selected clinics in October 2011, with two reminders at 1-week intervals sent to non-respondents. A covering letter explained the purpose of the survey, and a definition of evidence-based practice was given at the start of the questionnaire. Follow-up data were collected in September 2014 by sending the questionnaire to all practitioners at the selected clinics, i.e., the clinics from the baseline population (*n* = 795). Four reminders at 1-week intervals were distributed by e-mail. The response rates were 66% (*n* = 560) in 2011 and 63% (*n* = 498) in 2014. For further information regarding respondents and non-respondents (see Table 1).

**Table 1:** Respondents and distribution of gender, age, and work experience within occupational health service (OHS) for the whole study group, as well as stratified by type of OHS organization and profession

	Respondents *N* (%)		Women *N* (%)		Mean age year (SD)		Work experience within OHS year (SD)		Non-respondents *N* (%)	
	2011	2014	2011	2014	2011	2014	2011	2014	2011	2014
Whole study group	560 (100)	498 (100)	386 (69)	381 (77)	52 (9)	52 (9)	13 (10)	12 (9)	291 (100)	297 (100)
Private external large OHS	95 (17)	169 (34)	67 (71)	138 (82)	51 (8.7)	51 (10)	13 (11)	12 (9)	76 (26)	138 (46)
Private external small OHS	144 (26)	104 (21)	109 (76)	86 (83)	52 (8.7)	52 (9)	14 (10)	13 (9)	55 (19)	67 (22)
Private in-house OHS	84 (15)	31 (6)	52 (62)	21 (68)	50 (10)	51 (9)	11 (8)	12 (11)	54 (19)	12 (4)
Municipality/county council in-house OHS	237 (42)	194 (39)	158 (67)	136 (70)	53 (9)	53 (8)	13 (10)	12 (9)	106 (36)	80 (27)
Physician	69 (12)	56 (11)	24 (35)	28 (50)	56 (6)	59 (7)	11 (9)	11 (8)		
Nurse	178 (32)	163 (33)	166 (93)	158 (97)	53 (7)	52 (9)	14 (10)	12 (10)		
Psychologist/behavioral therapist	97 (17)	99 (20)	66 (68)	72 (72)	50 (10)	50 (10)	9 (6)	8 (6)		
Occupational therapist/physiotherapist	84 (15)	85 (17)	62 (74)	60 (70)	49 (9)	51 (10)	14 (10)	13 (9)		
Health instructor	18 (3)	13 (3)	14 (78)	12 (92)	41 (12)	45 (9)	9 (8)	7 (3)		
Work environment engineer	35 (6)	22 (4)	3 (9)	4 (18)	56 (10)	57 (7)	22 (11)	22 (9)		
Other	79 (14)	55 (11)	51 (64)	42 (76)	52 (8)	53 (8)	11 (9)	13 (9)		

#### Study population—interviews

Since management support is a well-known facilitator for implementation (cf. Hugenholtz et al. 2009), 105 managers from the OHS companies in the sample obtained in the first part of the study were invited to take part in a telephone interview. A strategic sampling guided the selection of managers. The inclusion criteria were that the participants should represent different kinds of ownership and work at least 50% of their time in a managerial position. Thirty of the invited managers were willing to be contacted for further information and 24 interviews took place, i.e., 23% of the invited managers.

## Data collection

### Survey questionnaire

The questionnaire was developed by the research group on the basis of earlier surveys of EBP or evidence-based medicine in occupational health settings (Heselmans et al. 2010; Schaafsma et al. 2004). The earlier surveys included questions about professional characteristics: demand for information in practice; information-seeking behavior; and attitudes towards evidence-based medicine or practice. This was supplemented by questions developed by the research group about organizational factors which influence opportunities for EBP. The items were reviewed by an OHS expert panel for face validity and the questionnaire was then revised through the consensus of the expert panel. The revised version was pilot-tested on a group of 25 employees from six different OHS companies. The group represented the OHS practitioners included in the study. The questionnaire was modified in accordance with the feedback from the pilot test, after which the expert group gave final approval to the questionnaire.

The final questionnaire consisted of 34 questions (nine background characteristics, 25 core area items). Fourteen questions were taken from Schaafsma et al. (2004) and had previously been translated into Swedish (Gummesson and Nordmark 2009). Two questions originated from Heselmans et al. (2010) and were translated by the research group, while nine questions were developed by the research group (see Appendix for the questionnaire). The questionnaire began with a definition of EBP (Sackett et al. 1996) and the participant’s characteristics. It assessed the following areas: the OHS practitioner’s interest in working according to EBP, and their need of and opportunities to do so (for the exact questions, see Table 2 footnote a); how the OHS practitioner sources information about the latest developments in his/her vocational field (Table 2, footnote b); organizational factors which influence opportunities to work according to EBP (Table 2, footnote c).

**Table 2:** Items with mean, standard deviation for baseline and follow-up and *p* values. Categorization of items into subscales based on exploratory factor analysis with parenthesized loading criteria. For subscales 1 and 2, a higher score indicates a more positive result. The opposite is the case for subscale 3

	Loading criteria	2011		2014		*P*-value
		Mean	SD	Mean	SD	
Subscale 1 Organizational competence, attitude and managerial support for evidence-based practice (ten questions)						
The OHS-unit where I’m employed encourages me to participate in research and development projects^c^	0.736	3.0	1.3	2.7	1.3	<0.001
The OHS-unit where I’m employed encourages me to participate in continuing education^c^	0.730	3.4	1.4	3.3	1.3	0.001
The OHS-unit where I’m employed encourages the purchases of professional literature in my field, such as textbooks^c^	0.725	3.7	1.3	3.3	1.3	<0.001
The management of the OHS where I’m employed encourages me to work according to EBP^c^	0.716	3.7	1.2	3.3	1.3	<0.001
At the OHS-unit where I’m employed we discuss methods and tools such as questionnaires, guidelines and regulations, risk assessment protocols^c^	0.678	3.9	1.1	3.6	1.1	<0.001
At the OHS-unit where I’m employed we discuss the best evidence base, for example we discuss the scientific literature^b^	0.561	3.4	1.2	3.3	1.1	NS
At the OHS-unit where I’m employed, there is sufficient expertise to work according to EBP^c^	0.538	4.0	0.9	3.5	1.0	<0.001
At the OHS-unit where I’m employed I am able to search and download research articles in my field from the Internet^c^	0.498	4.1	1.2	3.5	1.3	<0.001
I have the support of my colleagues to work according to EBP^a^	0.438	4.2	1.0	4.1	1.0	NS
I am able to acquire new knowledge by participating in research and development projects^b^	0.404	2.4	1.3	2.4	1.2	NS
Subscale 2 Implementation and development of evidence-based practice (five questions)						
Working according to EBP will become more important for occupational health services in the future^a^	0.632	4.6	0.9	4.4	0.8	<0.001
It is important for our corporate clients that the OHS-unit where I’m employed works in an evidence-based way^c^	0.570	4.1	1.0	3.9	1.0	<0.001
I need further training in how to work according to EBP in my professional field in the OHS^b^	0.495	4.3	0.9	4.1	0.9	<0.001
EBP is an integral part of my work^a^	0.409	4.2	0.9	4.1	0.9	NS
I regularly search the Internet for occupational health information^b^	0.402	4.0	1.1	3.9	1.0	NS
Subscale 3 Lack of knowledge and skills regarding evidence-based practice (four questions)						
I don’t have/I lack sufficient knowledge to critically examine and interpret evidence-based information^a^	0.731	2.7	1.2	2.6	1.1	NS
I don’t have/I lack sufficient knowledge and skills to use and implement evidence-based information^a^	0.704	2.7	1.2	2.5	1.1	NS
I need training in how to formulate questions for the effective retrieval of information from the Internet^b^	0.582	3.1	1.3	2.8	1.2	0.002
The reliability of the information I find in my professional field is low^b^	0.474	2.2	1.0	2.4	0.9	<0.001
Single items with loading criterion <0.4						
The use of EBP during consultations represents an extra workload^a^	0.201	2.9	1.3	2.7	1.0	0.002
The field of knowledge of my expert field is too wide to justify working according to EBP^a^	−0.264	2.8	1.3	2.8	1.0	NS
I ask colleagues for advice, for example about decision-making^b^	0.223	4.1	1.0	3.9	1.0	<0.001
I regularly search for information within my professional field in textbooks, journals, etc.^b^	0.378	3.9	1.0	3.8	1.0	NS
I do not expect to find satisfactory answers to my occupational health questions, for example in the scientific literature^b^	0.393	1.9	1.1	2.0	1.0	0.001
Lack of time is a barrier to my looking for occupational health information^b^	−0.309	2.9	1.2	3.0	1.1	NS

^a^The OHS practitioner’s interest in working according to EBP, and their need of and opportunities to do so

^b^How the OHS practitioner obtains new knowledge from their vocational field

^c^Organizational factors influencing opportunities to work according to EBP

Participant characteristics were obtained by means of questions about age, sex, years of work experience, type of position, education and occupational health service profession. OHS practitioners’ interest in working according to EBP, as well as their need and opportunity to do so, were assessed by questions about their views of EBP, collegial support, education and training in EBP practice and the use of EBP in the OHS practice. Obtaining information about the latest developments in their vocational field was measured by questions about information-seeking strategies and activities, training in search strategies and critical appraisal of evidence, and whether they were involved in research and development. To gauge the influence of organizational factors on opportunities to work according to EBP, participants were asked about managerial support for and encouragement of EBP at their OHS clinic and whether they had the necessary expertise to work with EBP. They were also asked about access to help with retrieving information from scientific databases or libraries, the purchase of medical literature, and whether their clinic facilitated participation in research and development projects. The response format was a Likert scale ranging from 1 to 5, with the anchors 1 = completely disagree, and 5 = completely agree. The participants were also asked to self-rate the top three facilitators and barriers with regard to EBP and there was an open text section for additional comments (the open text responses are not reported in this paper).

### Qualitative interviews

Semi-structured telephone interviews with open-ended questions were conducted between November 2014 and January 2015. The interviews were conducted by five occupational health nurses and one psychologist, all with prior training in qualitative methods and interview techniques. The interview guide addressed: the use of EBP at the OHS clinic; the OHS practitioner’s competence for working with EBP; staff recruitment strategies; client companies’ interest in and attitudes towards EBP; and barriers to and facilitators of EBP. The interviews lasted 20–45 min, were digitally recorded and transcribed verbatim.

### Statistical analysis

The data from the questionnaire were analyzed by using SPSS version 22.0. The demographic characteristics of the participants are presented as descriptive statistics. Differences between baseline and follow-up were analyzed using Mann–Whitney test. All results were considered significant at *P* < 0.05.

Differences between the baseline and follow-up data in terms of type of OHS organization (private external ≥50 OHS clinics; private external <50 OHS clinics; private in-house; municipality/county council in-house), age, profession and work experience were analyzed using *t* test.

To be able to construct indices based on the 25 core area items in the questionnaire, the quantitative analysis was carried out in three steps. Initially, ordinal exploratory factor analysis using Kendall’s tau-b was carried out on all 25 core area items in order to investigate the factor structures, the multidimensionality and the distribution of the items in the subscales. A loading criterion of 0.40 was applied and considered as the threshold for acceptance. On the basis of the analysis, 19 items could be grouped into three subscales, whilst the remaining six items that did not meet the loading criterion 0.40 were kept as single items (Table 2).

For each subscale, an index was constructed on a total score which was calculated by adding the scores of the items and dividing the result by the number of items included in the index. Where internal responses were missing, data were imputed. The criterion for this was that there should be answers to at least 70% of the items included in the subscale.

### Qualitative analysis

The data collected in the interviews were analyzed by means of inductive qualitative content analysis (Graneheim and Lundman 2004). The transcribed interviews were read through in order to gain an overview of the content. After this the coding process began and shorter segments of the data and key concepts of sentences addressing the aim of the study were identified. Each segment was labeled with a code summarizing the content. In the next step, the codes were compared and codes with a similar content were sorted into preliminary categories. After this, the emerging categories were reviewed, descriptions of the content of each category were written and the categories were given headings. Each category describes different aspects of and patterns in the data. The analysis was performed by the first author (EBB), with continuous support from the second author (TN). In the latter phase of analysis, the emergent categories were discussed by all authors and revised on the basis of these discussions. Quotations are used to illustrate the relationship between data and the categorization in order to increase the study’s transparency.

## Results

### Quantitative results

The response rate was 66% (*n* = 560) at baseline and 63% at follow-up (*n* = 498). The response rate differed between the four OHS categories. The profession with the highest proportion of women was occupational nurses (93 and 97%, respectively). Nurses accounted for the largest proportion of respondents at both time-points (32 and 33%, respectively). Men dominated among work environment engineers (89 and 82%). The mean age of the respondents was 52 years (SD 8.9) and the respondents had an average of 13 years’ (range 1–37) OHS work experience (Table 1).

### Factor analysis

Three subscales were constructed on the basis of the exploratory factor analysis (Table 2). Ten questions were classified into the subscale “Organizational competence, attitude and managerial support for EBP”. Five questions were classified into the subscale “Implementation and development of EBP”. Four questions were classified into the subscale “Lack of knowledge and skills regarding EBP”. In the subscales “Organizational competence, attitude and managerial support for EBP” and “Implementation and development of EBP”, a higher score indicates better competence and attitudes towards EBP; in subscale “Lack of knowledge and skills regarding EBP”, a lower score indicates better knowledge and skills.

### Organizational competence, attitude and managerial support for EBP

The results of the subscales from 2011 to 2014 are presented in Table 3. For the total sample and the subscale organizational competence, attitude and managerial support for EBP, a significant decrease (mean diff. 0.28; *p* < 0.001; 95% CI 0.18; 0.38) was found, which decreased from 3.6 to 3.3 between the two time-points. When the analyses were stratified according to type of OHS organization, the municipality/county council in-house OHS was the only OHS category with significantly lower point estimates at follow-up. For the single items included in the subscale (Table 2), a large proportion of the respondents reported high collegial support for EBP at both time-points. However, other items related to organizational factors significantly decreased between baseline and follow-up. Where managerial support for EBP was concerned, the respondents perceived less encouragement and support at follow-up. Respondents were also less likely to agree at follow-up that their OHS clinic had the necessary competence to practice EBP. Fewer workplace discussions about, for example, choice of methods and tools, were reported at follow-up than at baseline.

**Table 3:** Comparisons between baseline and follow-up on the three subscales

	2011		2014		Mean diff.	*P*-value (95% CI)
	Mean	SD	Mean	SD		
Subscale 1 Organizational competence, attitude and managerial support for evidence-based practice						
Whole study group	3.6	0.8	3.3	0.8	0.28	<0.001 (0.18; 0.38)
Private external large OHS	3.0	0.7	2.8	0.7	0.13	NS
Private external small OHS	3.3	0.8	3.2	0.7	0.13	NS
Private in-house OHS	3.7	0.8	3.4	0.7	0.30	NS
Municipality/county council in-house OHS	3.9	0.7	3.7	0.7	0.18	0.01 (0.04; 0.31)
Profession						
Work environment engineer	3.4	0.7	3.2	0.7	0.16	NS
Occupational therapist/physiotherapist	3.4	0.8	3.1	0.8	0.28	0.017 (0.05; 0.51)
Psychologist/behavioral scientist	3.4	0.9	3.1	0.9	0.31	0.014 (0.06; 0.56)
Physician	3.6	0.9	3.1	0.9	0.39	0.014 (0.08; 0.70)
Nurse	3.7	0.8	3.3	0.7	0.32	<0.001 (0.15; 0.50)
Health instructor	3.5	0.7	3.2	0.9	0.22	NS
Subscale 2 Implementation and development of evidence-based practice						
Whole study group	4.2	0.5	4.0	0.5	0.16	<0.001 (0.10; 0.22)
Private external large OHS	4.1	0.5	4.0	0.5	0.21	0.001 (0.08; 0.34)
Private external small OHS	4.1	0.5	4.0	0.6	0.16	0.033 (0.01; 0.31)
Private in-house OHS	4.2	0.6	4.2	0.5	0.01	NS
Municipality/county council in-house OHS	4.3	0.5	4.1	0.5	0.11	0.017 (0.02; 0.21)
Profession						
Work environment engineer	4.2	0.6	3.9	0.7	0.31	NS
Occupational therapist/physiotherapist	4.1	0.4	4.1	0.5	0.08	NS
Psychologist/behavioral scientist	4.1	0.5	4.0	0.6	0.15	NS
Physician	4.3	0.4	4.2	0.4	0.10	NS
Nurse	4.2	0.5	4.1	0.5	0.14	0.014 (0.02; 0.26)
Health instructor	4.3	0.5	4.3	0.4	−0.02	NS
Subscale 3 Lack of knowledge and skills regarding evidence-based practice						
Whole study group	2.7	0.9	2.6	0.7	0.11	0.02 (0.01; 0.21)
Private external large OHS	2.7	0.9	2.6	0.8	0.08	NS
Private external small OHS	2.8	0.9	2.6	0.7	0.20	NS
Private in-house OHS	2.7	0.8	2.5	0.8	0.17	NS
Municipality/county council in-house OHS	2.6	0.8	2.5	0.7	0.09	NS
Profession						
Work environment engineer	2.6	0.7	2.7	0.7	−0.07	NS
Occupational therapist/physiotherapist	2.7	0.9	2.6	0.7	0.10	NS
Psychologist/behavioral Scientist	2.4	0.9	2.3	0.8	0.12	NS
Physician	2.7	0.8	2.7	0.7	0.06	NS
Nurse	2.8	0.9	2.7	0.7	0.14	NS
Health instructor	2.8	0.8	3.0	0.6	−0.24	NS

Results for the whole study group, stratified on type of occupational health service (OHS) organization and profession. For subscales 1 and 2, a higher score indicates a more positive result. The opposite is the case for subscale 3

*NS* non-significant

### Implementation and development of EBP

For the subscale Implementation and development of EBP, the comparison between the two time-points revealed a significant decrease for the whole study group from 4.2 to 4.0 (mean diff. 0.16; *p* < 0.001; CI 0.10; 0.22). Respondents largely agreed that EBP was part of their work, with mean scores at baseline and follow-up of about 4. In line with addressing EBP as a part of their routine work, a significant reduction in educational needs was reported, indicating that the respondents may have improved their EBP-related skills during the follow-up. Where acquiring new knowledge was concerned, the respondents were less likely to agree that they needed to find information on the Internet at follow-up. They also agreed slightly less than at baseline that EBP will become more important for the OHS.

### Lack of knowledge and skills regarding EBP

For the subscale lack of knowledge and skills regarding EBP, the comparison over time showed a significant improvement in EBP knowledge and skills for the study group as a whole—from 2.7 to 2.6 (i.e., the lower the better) (mean diff. 0.11; *p* 0.02; 95% CI 0.01; 0.21). All types of OHS had significantly better scores in 2014 than in 2011, except for private in-house OHS. A significant change was demonstrated for the single item about the reliability of information, which implies that the respondents considered the reliability of information in their professional field as lower than before.

### Qualitative results

Of the 24 participants, 20 were women. Ten participants were employed at private external OHS and 14 at private in-house OHS. The mean age was 54 (min 37–max 68), the participants had between one and 20 years of managerial work experience in OHS settings (mean 5 years), and the following backgrounds: nurse (*n* = 6), physiotherapist/ergonomist (*n* = 6), psychologist/behavioral therapist/social worker (*n* = 4), work environment engineer (*n* = 3), biomedical scientist (*n* = 1), physician (*n* = 1), and unknown (*n* = 3). The results are presented under the headings of the identified categories.

The interviews revealed a generally positive attitude to EBP on the part of the OHS managers. EBP was perceived as giving the OHS the opportunity to perform interventions and treatments based on high quality and the best evidence. However, the managers regarded the implementation of EBP first and foremost as an issue for the OHS practitioners with an interest in EBP. Lack of time and resources and the contracts between the OHS and their corporate clinics were perceived as barriers to the development and use of EBP. The managers did not relate the reduction in use of EBP to the lack of organizational or managerial support for the OHS practitioners.

### The OHS practitioner’s interest is the driving force for EBP

The OHS managers regarded the OHS practitioners’ interest in EBP as the driving force for EBP and as an important facilitator for the use of EBP at the OHS clinics. In terms of organizational support, the managers expressed the view that it is the OHS practitioner’s own responsibility to keep up-to-date in their professional field. The OHS managers emphasized that enthusiastic OHS practitioners facilitated EBP at the OHS clinics by passing on information about the latest developments to their colleagues, for example guidelines or scientific literature. The OHS managers saw their own role as supporting the practitioners, giving them opportunities for sufficient training, participating in conferences or developing clinical guidelines. They saw continuity among the OHS practitioners as a facilitator. Colleagues commonly stopped working according to EBP if the OHS practitioner who had been the driving force for EBP at the clinic left. This was exemplified by one of the participants:

“In my opinion, we have time intended for EBP, weekly meetings with follow-up to make sure that there are good opportunities for EBP. We have a structure for this and a physician who’s in charge, who’s the driving force for EBP. If a person like that leaves the clinic, we unfortunately lose direction. EBP is dependent on the individual OHS practitioner.” (IP 11).

### Easy-to-use guidelines facilitate EBP

The OHS managers reported that little time and few resources were set aside for their practitioners to develop their own evidence-based guidelines. They therefore called for short, easy-to-use and cost-effective guidelines to facilitate EBP. They also commented that some guidelines were too comprehensive and that corporate clients were not willing to pay for EBP-based treatment, even if it was the recommended one. One participant gave the following description:

“We have cognitive behavioral therapists; they do not have the time to perform evidence-based therapy. As an example, we offer interpersonal therapy which is manual-based and one of the few therapies which is supported by evidence. But a well-adapted therapy involves about 16 h. It would be rare for a client company to pay for these sessions.” (IP 3).

### The contracts between the OHS and the corporate clients influence whether it is possible to work according to EBP

All OHS managers said that their clients expected the OHS to keep up-to-date and to offer high-quality services based on evidence. The managers felt that they did address issues related to best practice clinical decision-making in order to bring about changes in future contracts. Even if they regarded EBP as important for the delivery of high-quality treatments, they did not see the use of EBP as a “sales argument”, due to the fact that results from evidence-based care can never be guaranteed. As one participant commented during the interview:

“It could be devastating if you think you know for sure what the best evidence-based treatment is and if you use that as an argument for selling the treatment. If the treatment doesn’t give the effects that the client company expected, it doesn’t matter whether you have used an evidence-based treatment or not.” (IP 14).

### Collaboration between OHS clinics and research and development (RD) units

Some of the OHS managers reported that their OHS units were in touch with RD-units (i.e., universities) and that these contacts were dealt with by OHS practitioners with a particular interest in EBP. The interface between the OHS and the RD-unit gave the OHS access to new evidence and the opportunity to participate in the development of evidence-based guidelines. Moreover, supported by the RD-units the OHS practitioners were offered training in evidence-based guidelines or methods. Some of the larger OHS companies had an in-house RD-unit, which assisted with the latest research findings and evidence-based methods.

## Discussion

The primary aim of the present study was to investigate whether the development of evidence-based practice in relation to attitudes, knowledge and use in the Swedish occupational health service improved during the first 3 years of the government initiative. Our aim was also to investigate facilitators of and barriers to the implementation of EBP and whether these changed during the follow-up period. Overall, the results reveal a slight deteriorating trend. They indicate that the respondents experienced a lower degree of organizational competence, attitude and managerial support for EBP after 3 years. The only positive trend was an improvement in knowledge about EBP. The follow-up period saw the establishment of a knowledge base and research infrastructure in the OHS.

### Methodological considerations

In the present study, quantitative data from the web-based questionnaires were combined with qualitative data from interviews. The use of more than one data source improves the validity of results (Padgett 2009).

The overall response rate of the questionnaire was 66% in 2011 and 63% in 2014, which can be seen as acceptable for a web-based survey (Draugalis et al. 2008). However, the non-response rate is a potential source of positive selection bias, as individuals with more interest and competence in EBP may be more likely to respond. Equally, since the study population was initially selected at OHS company level, the same could hold for the OHS companies that chose to participate in the study—in other words, companies more interested in EBP may have been more likely to accept the invitation. However, even if this may have affected the magnitude of the scores it should not have affected the magnitude of the change over time, which is the focus of this study. Another shortcoming is that the participating OHS companies differed slightly between baseline and follow-up. However, we performed additional analyses (not shown) only using the OHS companies that had answered at both baseline and follow-up. These additional analyses did not result in any differences.

Most of the questions were based on previous questionnaire studies (Gummesson and Nordmark 2009; Heselmans et al. 2010; Schaafsma et al. 2004), but were modified to fit the study population. However, the definition of EBP can to some extent be considered context-specific. A fairly general definition of EBP was given at the beginning of the questionnaire. Given that the respondents represented a variety of occupations from a variety of disciplines (i.e., medicine, social sciences and engineering), the term “evidence” may have been interpreted differently depending on the respondent’s professional background (Satterfield et al. 2009). However, questions about the individual’s capacity to perform EBP might be too intricate to answer, thus there is a risk of social-response bias in the quantitative and qualitative data. The questions used in the interviews were designed to support the participants’ reflections on the use of EBP within their OHS clinics. The credibility of the qualitative findings was ensured by keeping the analysis process close to the interview data and the emerging analysis was continuously discussed by the research group (Tong et al. 2007).

In general, the changes over time were small. Even if several of the analyses were statically significant it is hard to draw conclusions regarding their practical significance with regard to EBP. The survey results should therefore be interpreted with some caution.

### Organizational competence, attitude and managerial support

The respondents reported slightly less organizational competence, attitude and managerial support for EBP in 2014 than in 2011. Previous research indicates that support from the management or the board is an important facilitator for the implementation of EBP (Weiner et al. 2011). For example, Preece et al. (2012) reported that organizations with boards that actively support EBP facilitated the introduction of guidelines (Preece et al. 2012). The qualitative findings confirmed the results of the questionnaire. The OHS managers saw EBP as the responsibility of individual practitioners and the latter’s interest in EBP was seen as the driving force. Some of the managers reported that they held meetings to disseminate research findings which were relevant for the implementation of EBP in the OHS. They also regarded lack of time and resources as potential barriers to EBP, in line with previous research (Joosen et al. 2015; Zwolsman et al. 2012). However, the decline in perceived support may also be explained by a more critical stance towards EBP among the respondents, resulting from improvements in practitioners’ attitudes towards and knowledge of what EBP actually is and what it involves.

### Knowledge and skills regarding EBP

The findings of our study reveal a positive trend in knowledge about EBP, even if the changes are small. In recent years, technological advances such as access to information on the Internet and the launch of the first OHS guideline have contributed to a rapidly increasing availability of knowledge and information, even as teaching and training methods have improved. The findings from the interviews revealed that the OHS managers regarded guidelines and collaboration with RD-units as facilitating EBP, since such collaboration implied access to education in EBP-guidelines and/or evidence-based methods. However, previous studies suggest that the effect of continuing education on compliance with desired practice is small (Forsetlund et al. 2009). To succeed in the sustainable implementation of EBP, training needs to be combined with workplace employer support and workplace change strategies (Novak and McIntyre 2010) which support change in behaviors.

### Implementation and development of EBP

The definition of EBP used in the questionnaire was quite general and not linked to the use of evidence-based guidelines or assessment protocols. Our findings demonstrate a slight decrease in positive attitudes towards the implementation and development of EBP. The point estimates at baseline and follow-up indicate that the respondents largely agreed with the items included in the subscale. Practitioners’ attitudes towards EBP are known to be a predictor of future use (O’Donnell 2004; Quiros et al. 2007). The slightly less positive attitude among the OHS practitioners at follow-up might be explained by lack of time, resources and staff turnover, as mentioned by the OHS managers. This is in line with previous studies on facilitating factors for sustained implementation (Lau et al. 2016).

When the analysis was stratified according to type of OHS organization, the results demonstrated significant differences in relation to the implementation and development of EBP for all types of OHS organizations except private in-house OHS. Previous research supports the notion that public sector organizations are more bureaucratic than private sector agencies, have more formal rules and regulations and hierarchical authority structures. These factors have been linked to a lower degree of interest in innovation and change (Aarons et al. 2009). Thus, public sector agencies are likely to provide less organizational support for the development and use of EBP.

In summary, the results indicate that EBP continues to interest OHS practitioners, even if they experienced slightly less organizational and managerial support during the 3-year follow-up. At both time-points the respondents demonstrated a positive attitude towards implementing EBP. However, they saw the relatively low levels of organizational support and skill development support as a barrier. The first 3-year period of the government initiative was characterized by passive dissemination of EBP and a lack of support and feedback for the OHS units. This might explain the study’s findings that OHS practitioners as well as OHS managers observed a lack of organizational and managerial support. In fact, it takes more than the drive of the individual practitioner to succeed in implementing EBP. In line with previous research, we therefore conclude that active implementation strategies which include support for management and organizations are needed in order to achieve a sustainable implementation of EBP among OHS practitioners and in OHS organizations. The next 3-year period should be accompanied by more active implementation strategies, such as the development of practice guidelines through a partnership model, seminars and workshops. It is also necessary to actively direct interventions at OHS company managements in order to improve the implementation of EBP within OHS. On the basis of our findings, we suggest that future research evaluates the active strategies for implementation that are used in the next 3-year period of the government initiative, in order to evaluate those factors which facilitate and hinder the implementation of EBP in OHS. Furthermore, future research should address issues related to senior management, their support for EBP and how EBP could be facilitated at different levels in the organizations.

## Conclusions

In general, one can say that OHS managers and practitioners are positive to EBP. However, the questionnaire and the interviews indicate that important cornerstones for the successful implementation and development of evidence-based practices in OHS are missing. EBP was seen as the responsibility of the individual practitioner rather than an organizational issue. Thus, the findings underscore the need to educate managers in how to support EBP at the organizational level by creating an infrastructure for EBP in the OHS.

## Appendix: Questionaire

### Questions from Schaafsma et al. (2004)

At the OHS-unit where I’m employed we discuss the best evidence base, for example we discuss the scientific literature.

I have the support of my colleagues to work according to EBP.

Working according to EBP will become more important for occupational health services in the future.

I need further training in how to work according to EBP in my professional field in the OHS.

EBP is an integral part of my work.

I regularly search the Internet for occupational health information.

I don’t have/I lack sufficient knowledge to critically examine and interpret evidence-based information.

I don’t have/I lack sufficient knowledge and skills to use and implement evidence-based information.

I need training in how to formulate questions for the effective retrieval of information from the Internet.

The field of knowledge of my expert field is too wide to justify working according to EBP.

I ask colleagues for advice, for example about decision-making.

I regularly search for information within my professional field in textbooks, journals etc.

I do not expect to find satisfactory answers to my occupational health questions, for example in the scientific literature.

Lack of time is a barrier to my looking for occupational health information.

### Questions from Heselmans et al. (2010)

The reliability of the information I find in my professional field is low.

The use of EBP during consultations represents an extra workload.

### Questions developed by the research group (translated into English for this manuscript)

The OHS-unit where I’m employed encourages me to participate in research and development projects.

The OHS-unit where I’m employed encourages me to participate in continuing education.

The OHS-unit where I’m employed encourages the purchases of professional literature in my field, such as textbooks.

The management of the OHS where I’m employed encourages me to work according to EBP.

At the OHS-unit where I’m employed we discuss methods and tools such as questionnaires, guidelines and regulations, risk assessment protocols.

At the OHS-unit where I’m employed, there is sufficient expertise to work in accordance with EBP.

At the OHS-unit where I’m employed I am able to search and download research articles in my field from the Internet.

I am able to acquire new knowledge by participating in research and development projects.

It is important for our corporate clients that the OHS-unit where I’m employed works in an evidence-based way.
